# O7.7. COGNITIVE FUNCTIONING FOLLOWING DISCONTINUATION OF ANTIPSYCHOTIC MEDICATION: A SUB-GROUP ANALYSIS FROM THE OPUS II TRIAL

**DOI:** 10.1093/schbul/sby015.236

**Published:** 2018-04-01

**Authors:** Nikolai Albert, Lasse Randers, Kelly Allott, Heidi Jensen, Marianne Melau, Carsten Hjorthøj, Merete Nordentoft

**Affiliations:** 1 Mental Health Centre Copenhagen; 2 Orygen, The National Centre of Excellence in Youth Mental Health; 3 Mental Health Centre Copenhagen, University of Copenhagen

## Abstract

**Background:**

The presence of cognitive defects in patients suffering from schizophrenia is well established. While the earlier “Kraepelinian” view was one of deteriorating cognitive functioning, more recent studies have found that cognitive deficits tend to be stable or improving over time. Cognitive impairments are associated with poorer functional outcomes and understanding the factors that influence cognitive functioning is critical for understanding how to improve cognitive and functional outcomes in patients. The effect of antipsychotics medication on cognitive functioning in patients diagnosed with schizophrenia is poorly understood. Some studies of second-generation antipsychotics indicated that they improved cognitive functioning while other studies have found that they decrease the level of cognitive functioning.

**Methods:**

We included patients with schizophrenia who were in treatment with antipsychotics 1.5 years (baseline) after initiation of treatment and followed them up 3.5 years later (n=189). At follow-up 60 (32%) had discontinued their antipsychotic treatment and 129 (68%) were still taking antipsychotics. Using the Brief Assessment of Cognition in Schizophrenia (BACS) we assessed cognition at baseline and follow-up.

**Results:**

The patients who had discontinued their medication had a higher level of cognitive functioning in all domains at baseline, as well as Global cognitive function (mean z-score -1.50 (SD 1.24) vs. -2.27 (SD 1.30), p<.001). After controlling for relevant confounders (age, sex, baseline functioning and negative symptoms) those who discontinued antipsychotic medication improved significantly more than those who remained on antipsychotic medication during the course of the follow-up on the Token Motor Task (estimated mean change difference -0.46, 95% CI(-0.89; -0.04), p=0.031), the Speed of Processing Domain (estimated mean change difference -0.38, 95% CI(-0.68; -0.08), p=0.012), and Global cognition (estimated mean change difference -0.36, 95% CI(-0.66; -0.07), p=0.016).

**Discussion:**

Due to the naturalistic design we cannot conclude on the direction of the relationship between antipsychotic medication and cognition. There is no evidence that discontinuation of medication had a negative effect on cognitive functioning. Rather, we find that that discontinuation of medication was associated with better cognitive functioning.

